# Relationship between tooth loss and tongue pressure and the effectiveness of removable denture for older people requiring long-term care

**DOI:** 10.1016/j.jds.2025.06.028

**Published:** 2025-07-31

**Authors:** Madoka Funahara, Yuki Sakamoto, Haruka Oyama, Sakiko Soutome

**Affiliations:** a School of Oral Health Sciences, Kyushu Dental University, Fukuoka, Japan; b Department of Dentistry and Oral Surgery, Kansai Medical University Medical Center, Osaka, Japan; c Funahara Dental Clinic, Hyogo, Japan; d Department of Oral Health, Nagasaki University Graduate School of Biomedical Sciences, Nagasaki, Japan

**Keywords:** Tongue pressure, Functional tooth unit, Prosthetic treatment, Older people, Salivary bacteria, Aspiration pneumonia

## Abstract

**Background/purpose:**

Aspiration pneumonia is a major health concern among older adults, particularly those requiring long-term care. It is often caused by silent aspiration of saliva-containing pathogenic bacteria and exacerbated by impaired oral and swallowing functions. Tongue pressure is a key factor in maintaining these functions, and its decline is associated with increased bacterial amount in the saliva and higher mortality. However, the relationship among tongue pressure, occlusal status, and prosthetic treatment in older individuals requiring care remains unclear. This study aimed to investigate the association between tooth loss and decreased tongue pressure in older individuals requiring long-term care, and to evaluate whether wearing removable dentures can mitigate this decline.

**Materials and methods:**

Fifty-three participants (mean age: 82.1 years) from three nursing facilities in Japan were assessed. The collected data included tongue pressure, number of teeth, functional tooth units (FTU), oral hygiene, tongue coating index, xerostomia, and salivary bacterial count. Bacterial counts were measured using real-time PCR.

**Results:**

Reduced tongue pressure was significantly correlated with increased bacterial counts in the saliva. Multivariate analysis confirmed the significant relationship between decreased nif-FTU levels and reduced tongue pressure. Although not statistically significant, denture use tended to preserve tongue pressure in patients with reduced natural dentition.

**Conclusion:**

Tooth loss may contribute to reduced tongue pressure and increased bacterial amount in the saliva of older adults. Prosthetic treatments, including dentures, may help maintain oral function and reduce the risk of aspiration pneumonia.

## Introduction

Aspiration pneumonia is a serious health problem among older adults, and its risk is particularly high among those who require nursing care. Aspiration pneumonia is caused by a combination of silent aspiration of saliva containing pathogenic microorganisms and a weakened immune system. Therefore, it is important to establish good oral hygiene and maintain oral functions such as swallowing.

The tongue is responsible for pushing food into the esophagus during swallowing, and maintaining tongue pressure is important for ma10intaining mastication and swallowing functions.^1^^,^^2^ Decreased tongue pressure increases the risk of food and saliva accidently entering the airway, leading to aspiration pneumonia. Tongue pressure decreases with advanced age and general frailty, and it has been reported that decreased tongue pressure not only reduces swallowing function but also increases the number of bacteria in the saliva,^3^ leading to increased mortality from aspiration pneumonia.^4^ Maintaining tongue pressure is important for healthy living in older people. However, factors related to decreased tongue pressure and how to maintain tongue pressure remain unclear.

Tashiro et al. reported that as the number of functioning molars decreases, tongue pressure also decreases. Further, they reported that fixed prosthetics with bridges or dental implants prevent tongue pressure reduction, but removable dentures do not have the power to control tongue pressure reduction.^5^ However, their study was conducted in healthy older adults, and no study has examined the factors associated with tongue pressure reduction and the effects of prosthetic treatment in older adult patients requiring long-term care.

This study aimed to determine whether tooth loss was associated with decreased tongue pressure in older adults requiring the long-term care and whether wearing removable dentures could prevent tongue pressure loss. This study suggests effective interventions for preventing aspiration pneumonia in older adults requiring long-term care.

## Materials and methods

### Study design and participants

The subjects of this study were older people aged 65 years or more who required long-term care, were residing in three older adult care facilities in Hyogo Prefecture, Japan, and whose consent for this study was obtained. Those who were able to walk on their own; received tube feeding; had poor comprehension; could not measure tongue pressure; and had neuromuscular diseases such as muscular dystrophy, Parkinson's disease, and amyotrophic lateral sclerosis were excluded.

### Factors examined

Sex, age, grip strength, number of teeth, functional tooth unit (FTU), denture use, oral hygiene status, tongue coating index, tongue pressure, xerostomia, and amount of bacteria in the saliva were investigated. Grip strength was measured twice with both hands using a digital grip strength meter (T.K.K. 5405; Takei Scientific Instruments Co.,Ltd., Niigata, Japan), and the highest value was recorded.^6^ FTU scores were defined according to the number of corresponding upper and lower bicuspids or molars as follows: the corresponding upper and lower bicuspids were assigned one point, and the corresponding upper and lower molars were assigned two points.^7^ Thus, if all bicuspids and molars were engaged, the FTU was defined as 12 points, which was calculated as nif-FTU, counted as the sum of natural teeth and fixed prosthetics (bridges and dental implants), and t-FTU, counted as teeth with removable dentures. Oral hygiene status was based on the debris index (DI) of the Oral Hygiene Index (OHI) and denture plaque buildup.^8^ A DI of less than two and little denture plaque is considered good, while a DI of more than two or high denture plaque considered poor. The tongue coating index was measured according to the method described by Shimizu.^9^ The dorsum of the tongue was divided into 9 sections, and the condition of tongue coating on each section was rated on a scale of 0–2, with the tongue coating index as the total score. A score of 0 was assigned if there was no tongue coating, 1 if the tongue papillae could be seen but the coating was thin, and 2 if the tongue coating was so thick that the tongue papillae could not be seen. Tongue pressure was measured using a JMS Tongue Pressure Measuring Device (JMS Co. Ltd., Hiroshima, Japan), which consisted of a probe and digital tongue depressometer with a balloon attached to the tip of the probe.^10^ Oral dryness was measured using the Challacombe Scale.^11^ If the dental mirror did not adhere to the buccal mucosa, the patient was considered to have no dryness. If there was little saliva and the dental mirror adhered to the buccal mucosa, or there was no saliva retention at the floor of the mouth, the patient was considered to be dry.

### Methods of sample collection and bacteria counting

A strip of filter paper (Advantec Nobuto Blood Sampling Paper strip type I; TOYOBO Co., Ltd., Osaka, Japan), 5 mm wide at the tip and 30 mm long, was placed on the floor of the mouth for 10 s, and 10 mm of the tip of the filter paper soaked with saliva was cut. DNA was extracted from the filter paper containing saliva, and the number of bacteria was quantified by real-time PCR using a universal primer targeting 16s ribosomal RNA. The reaction conditions and other details have been reported previously.^12^^,^^13^

### Statistical analysis

All the statistical analyses were performed using SPSS version 26 (IBM Japan, Ltd., Tokyo, Japan). Factors related to bacterial counts and tongue pressure were analyzed using the Mann–Whitney U test or Kruskal–Wallis test for categorical variables, Spearman's rank correlation coefficient for continuous variables for univariate analysis, and multiple regression analysis for multivariate analysis. Statistical significance was set at *P* < 0.05.

### Ethics and registration

The study was conducted between September and October 2021. This study conformed to the tenets of the Declaration of Helsinki. Ethical approval was obtained from the Institutional Review Board of Nagasaki University Hospital (#21091314). Informed consent was obtained from all the patients. The study was registered with the University Hospital Medical Information Network Clinical Trials Registry (UMIN-CTR) (#UMIN000045466; September 21, 2021).

## Results

### Participants’ characteristics

Fifty-three patients were enrolled in the study. The patients included 17 men and 36 women, with a mean age of 82.1 years. The mean grip strength and tongue pressure were 9.44 kg and 12.0 kPa, respectively, and most of them showed low values. The number of remaining teeth was 11.9, and the nif-FTU was greater than 8 in 14 patients, and less than 8 in 39 patients (Table 1).

**Table 1:** Characteristics of participants.

Variable		Number/mean ± SD
Age	(years)	82.1 ± 10.8
Sex	Man	17
	Woman	36
Hand grip	(kg)	9.44 ± 8.25
Tongue pressure	(kPa)	12.0 ± 10.9
Tongue coating index		3.92 ± 3.92
Denture use	(−)	24
	(+)	29
Oral hygiene	Good	14
	Bad	39
Dry mouth	(−)	39
	(+)	14
Number of teeth		11.9 ± 10.5
nif-FTU	0–7	39
	8–12	14

Abbreviations.

SD: standard deviation.

nif-FTU: natural teeth and artificial teeth from fixed prostheses or implant-supported functional tooth unit.

### Number of total bacteria in saliva and related factors

The median (25, 75 percentile) logarithm of the total bacterial count in samples extracted from the filter paper was 3.89 (3.35, 4.56). Univariate analysis of the factors associated with bacterial counts showed that tongue pressure (*P* = 0.012) was significantly associated with an increased bacterial count (Table 2). In the multivariate analysis, tongue pressure (*P* = 0.022) and dry mouth (*P* = 0.036) were identified as factors associated with salivary bacterial counts (Table 3).

**Table 2:** Factors related to the number of total bacteria in saliva (univariate analysis).

Variable			
i) Categorical data		Logarithm of number of bacteria	P-value

Sex	Man	3.95 ± 0.76	0.558
	Woman	4.11 ± 1.01	
Denture use	(−)	4.16 ± 1.16	0.487
	(+)	3.98 ± 0.71	
Oral hygiene	Good	3.75 ± 0.96	0.149
	Bad	4.17 ± 0.91	
Dry mouth	(−)	3.90 ± 0.79	0.034
	(+)	4.51 ± 1.16	
nif-FTU	<8	4.12 ± 0.97	0.329
	≥8	3.80 ± 0.75	

ii) Continuous data		Spearman's correlation coefficient	P-value

Age (years)		0.163	0.244
Hand grip (kg)		−0.159	0.255
Tongue pressure (kPa)		−0.344	0.012
Tongue coating index		−0.062	0.660
Number of teeth		−0.042	0.764

Abbreviations: nif-FTU: natural teeth and artificial teeth from fixed prostheses or implant-supported functional tooth unit.

### Factors related to the tongue pressure

Univariate analysis of factors related to tongue pressure showed significant associations with nif-FTU (*P* = 0.011) and tongue coating index (*P* = 0.034) (Table 4). When these two factors were included as covariates in the multivariate analysis, tongue pressure was significantly higher when nif-FTU was greater than 8 than when nif-FTU was less than 7 (*P* = 0.016) (Table 5).

**Table 3:** Factors related to the number of total bacteria in saliva (multivariate anaysis).

	Unstandardized coefficients		Standardized coefficients	95 % confidence interval		P-value
	B	SE	β	Lower	Upper	
Dry mouth	0.579	0.269	0.277	0.039	1.118	0.036
Tongue pressure (kPa)	−0.026	0.011	−0.304	−0.048	−0.004	0.022

**Table 4:** Factors related to the tongue pressure (univariate analysis).

Variable			
i) Categorical data		Logarithm of number of bacteria	P-value

Sex	Man	12.2 ± 12.9	0.938
	Woman	11.9 ± 10.5	
Denture use	(−)	12.9 ± 12.0	0.596
	(+)	11.3 ± 9.97	
Oral hygiene	Good	13.1 ± 13.2	0.667
	Bad	11.6 ± 10.0	
Dry mouth	(−)	12.3 ± 11.3	0.730
	(+)	11.2 ± 9.94	
nif-FTU	<8	10.2 ± 9.25	0.011
	≥8	19.8 ± 14.1	

ii) Continuous data		Spearman's correlation coefficient	P-value

Age (years)		−0.124	0.378
Hand grip (kg)		0.195	0.161
Tongue coating index		0.292	0.034
Number of teeth		0.115	0.412
			

Abbreviations: nif-FTU: natural teeth and artificial teeth from fixed prostheses or implant-supported functional tooth unit.

**Table 5:** Factors related to the tongue pressure (multivariate anaysis).

	Unstandardized coefficients		Standardized coefficients	95 % confidence interval		P-value
	B	SE	β	Lower	Upper	
nif-FTU	8.929	3.581	0.325	1.736	16.122	0.016
Tongue coating index	0.563	0.361	0.203	−0.162	1.288	0.125

Abbreviations: nif-FTU: natural teeth and artificial teeth from fixed prostheses or implant-supported functional tooth unit.

### Relationship between tongue pressure and t-FTU

**Figure 1:**
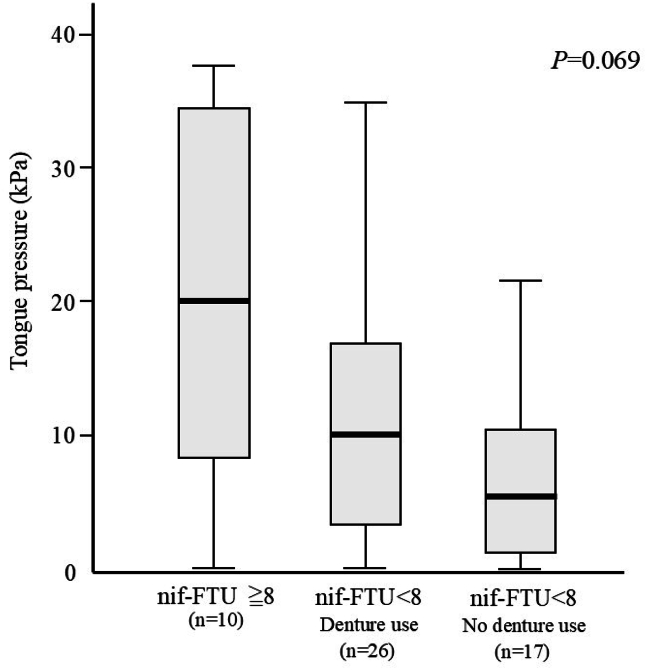
Relationship between nif-FTU and tongue pressure. Participants with a nif-FTU of 8 or more had higher tongue pressure than those with a nif-FTU of less than 8. However, even among those with a nif-FTU of less than 8, the use of removable dentures tended to suppress the decline in tongue pressure.

To investigate the effect of prosthetic treatment on tongue pressure maintenance, the relationship between tongue pressure and t-FTU was examined. Even though the nif-FTU was less than 7, those with dentures tend to maintain tongue pressure better than those without dentures. However, the difference was not significant (*P* = 0.069), possibly because of the small number of cases (Fig. 1).

## Discussion

Aspiration pneumonia is caused by aspiration of saliva-containing bacteria and decreased systemic resistance. Various preventive measures have been taken to reduce salivary bacteria to prevent aspiration pneumonia. Since bacteria in dental plaque are found in the lungs of patients with aspiration pneumonia, it was thought that the causative organisms of aspiration pneumonia were derived from the dental plaque.^14^, ^15^, ^16^ Therefore, recommended oral care is centered on plaque removal.^17^, ^18^, ^19^, ^20^, ^21^, ^22^ However, because dentulous and edentulous jaws have similar rates of aspiration pneumonia, the theory that dental plaque is the primary cause of aspiration pneumonia remains questionable.^23^

We have previously reported several factors associated with increased bacterial counts in saliva. We examined the differences in salivary bacterial counts in healthy subjects with high and low plaque build-up and found no statistical differences between the two groups. A study of older adult patients with outpatient dental visits showed that the factors affecting the number of bacteria in the saliva included a small number of remaining teeth and poor oral hygiene.^24^ In a study of older adults admitted to a facility who require long-term care and eat orally, poor oral hygiene, advanced age, and decreased tongue pressure were identified as factors associated with increased bacterial counts in the saliva.^3^ In a study of older adults admitted to a facility for the older adults requiring nursing care, including gastroduodenal patients, the following factors were identified: decreased food morphology (swallowing function) and inability to rinse the mouth (oral function). Thus, there was no agreement on the factors related to the number of bacteria in the saliva depending on the subject's situation. These results suggest that the number of bacteria in saliva is also influenced by the state of oral function. In healthy individuals, even if the number of bacteria in saliva temporarily increases, the number of bacteria in saliva does not increase beyond a certain level because the bacteria are frequently swallowed and new saliva is secreted. However, if the number of bacteria in the saliva decreases owing to poor oral and swallowing functions, the number of bacteria in the saliva will increase. Based on the results of previous studies, oral hygiene is thought to influence the number of bacteria in saliva. Furthermore, in older adult patients who are no longer able to consume food orally, oral and swallowing dysfunction are strongly related to an increase in salivary bacterial counts, and other factors such as oral hygiene may no longer be significant factors.

As described above, an increase or decrease in the number of bacteria in saliva is influenced by the self-cleaning function of the oral cavity, and the self-cleaning function is influenced by oral and swallowing functions. Swallowing is performed by coordinating various oral parts. The tongue has diverse functions such as assisting in the crushing of food during mastication, forming food clumps, and sending food clumps into the esophagus during swallowing.^25^, ^26^, ^27^, ^28^ Therefore, tongue pressure is related to oral and swallowing functions.^29^ In our previous study of ambulatory older people, factors associated with low tongue pressure included advanced age, decreased grip strength, and decreased nif-FTU.^5^ The reason that nif-FTU was listed as a factor related to tongue pressure was thought to be that the presence of chewing teeth maintained the intake of solid food and prevented oral functional decline by providing natural training to eat a loaded meal three times a day. This previous study reported that restoring occlusion with fixed prosthetic devices such as bridges or dental implants prevented the decrease in tongue pressure, whereas restoring occlusion with removable dentures did not prevent the decrease in tongue pressure. In the present study, which was conducted on older people requiring long-term care, there was a tendency for tongue pressure to improve in denture wearers compared to those without dentures, even when the nif-FTU was less than 8. However, the difference was not significant (*P* = 0.069), possibly because of the small number of cases.

To date, the number of bacteria in saliva is thought to be associated with oral hygiene. Oral care has been used to reduce the number of bacteria, and oral function and swallowing training have focused on preventing aspiration. However, the results of this study indicate that oral function and swallowing training not only prevent aspiration, but also reduce the number of bacteria in the saliva. Furthermore, the maintenance of occlusion allows the perioral muscles and tongue to ingest solid food, which may contribute to the maintenance and improvement of oral function. These findings suggest that prosthetic treatment after tooth loss may reduce the number of bacteria in saliva and prevent aspiration pneumonia.

This study had several limitations. This was a cross-sectional study with a small number of cases; thus, it is unclear whether the results obtained can be generalized. Although the effectiveness of dentures has been suggested, the small number of cases did not result in significant differences, and the denture fit and occlusal force were not examined. Nevertheless, this is the first study to suggest that in older adult patients requiring long-term care, a decrease in the number of teeth contributing to occlusion may lead to a decrease in tongue pressure, which in turn may lead to an increase in the number of bacteria in the saliva; moreover, prosthetic treatment may be able to suppress these effects. We plan to increase the number of cases to be studied in the future and examine changes before and after prosthetic treatment through a longitudinal study.

In conclusion, the association between bacterial numbers in the saliva, tooth loss, and tongue pressure was investigated in 53 older individuals requiring long-term care. Reduced tongue pressure was significantly correlated with increased bacterial counts in the saliva. Multivariate analysis confirmed the significant relationship between decreased nif-FTU levels and tongue pressure. Prosthetic treatments, including dentures, may help maintain tongue pressure and reduce the risk of aspiration pneumonia.

## Declaration of competing interest

The authors have no conflicts of interest relevant to this article.
